# In Vitro/In Vivo Hepatoprotective and Antioxidant Effects of Defatted Extract and a Phenolic Fraction Obtained from *Phlomis Tuberosa*

**DOI:** 10.3390/ijms241310631

**Published:** 2023-06-25

**Authors:** Magdalena Kondeva-Burdina, Aleksandar Shkondrov, Georgi Popov, Vasil Manov, Ilina Krasteva

**Affiliations:** 1 Laboratory of Drug Metabolism and Drug Toxicity, Department of Pharmacology, Pharmacotherapy and Toxicology, Faculty of Pharmacy, Medical University of Sofia, 2 Dunav st., 1000 Sofia, Bulgaria; 2 Department of Pharmacognosy, Faculty of Pharmacy, Medical University of Sofia, 2 Dunav st., 1000 Sofia, Bulgaria; shkondrov@pharmfac.mu-sofia.bg (A.S.); ikrasteva@pharmfac.mu-sofia.bg (I.K.); 3 Department of Non-infectious Diseases, Pathology and Pharmacology, Faculty of Veterinary Medicine, University of Forestry, 1000 Sofia, Bulgaria; georgistpopov@yahoo.com (G.P.); manov_vet@abv.bg (V.M.)

**Keywords:** *Phlomis tuberosa* extract and phenolic fraction, in vitro/in vivo study, isolated rat hepatocytes, rats, hepatoprotection, histopathology

## Abstract

An in vitro/in vivo hepatotoxicity and hepatoprotection evaluation of a defatted extract and a phenolic fraction from *Phlomis tuberosa*, administered alone and in a carbon tetrachloride (CCl_4_)-induced metabolic bioactivation model, was performed. The extract and the phenolic fraction were analysed by high performance liquid chromatography (HPLC) to determine the total flavonoid content, to identify flavonoids and to quantify verbascoside. In addition, total polyphenolics in the samples were expressed as gallic acid equivalents. Applied alone, the extract and the fraction (5, 10 and 50 µg/mL) did not show a statistically significant hepatotoxic effect on isolated rat hepatocytes in vitro. In a CCl_4_-induced hepatotoxicity model, the samples exhibited a concentration-dependent, statistically significant hepatoprotective effect, which was most pronounced at 50 µg/mL for both. The phenolic fraction exhibited a more pronounced hepatoprotective effect compared to the extract. Data from the in vitro study on the effects of the extract were also confirmed in the in vivo experiment conducted in a CCl_4_-induced hepatotoxicity model in rats. A histopathological study showed that the animals treated with CCl_4_ and the extract had an unaltered histoarchitecture of the liver. The effects of the extract were the same as those of silymarin.

## 1. Introduction

The main biotransformation processes related to the deactivation, bioactivation and detoxification of various xenobiotics take place in the liver. There are several in vivo and in vitro methodological setups to evaluate this process. Isolated hepatocytes (as a suspension or in a culture) are widely used in experimental biochemistry, pharmacology and toxicology. After isolation and incubation under suitable conditions, hepatocytes retain their main function in vitro and, accordingly, the biochemical processes associated with phases I and II of biotransformation. Isolated rat hepatocytes are a stable system that can be used in various in vitro studies for cytotoxicity, metabolism, etc., of new promising compounds of synthetic and natural origin. One of the main criteria adopted and recommended by the European Centre for the Validation of Alternative Methods (ECVAM) that is used to assess the functional–metabolic status of hepatocytes is: the viability (determined by the trypan blue test) and the release of the lactate dehydrogenase enzyme (in cases of cell membrane impairment). The toxic changes that occur in the cell at different levels, because of biotransformation and/or bioactivation of the investigated substances, can be evaluated by a change in the level of the tripeptide glutathione (one of the most important endogenous factors involved in cell protection), as well as a change in the amount of malondialdehyde (the product formed from the lipid peroxidation).

There are more than 20,000 species of plants used in traditional medicine, which are also potential raw materials for the discovery of new medicines [1]. With the advancement of modern medicine and drug research, chemical synthesis has replaced plants as the main source of drugs in industrialized countries. However, most of the world’s population cannot afford pharmaceuticals and therefore use plant-based medicines. Traditional medicinal plants have received considerable attention because their bioactive components can lead to the development of new medicinal products [1].

*Phlomis* is a large genus of plants in the *Lamiaceae* family, with over 100 species distributed across the continents of Europe, Asia and North Africa. In the species, the main groups of secondary metabolites are: flavonoids, phenylethanoids (mainly verbascoside), iridoids, essential oil, etc. [2]. Many flowering aerial parts of the *Phlomis* species are applied as a herbal tea to treat gastrointestinal problems and to protect the liver, kidneys, bones and cardiovascular system. A lot of in vitro studies have been performed on extracts or isolated compounds from representatives of the genus. Antioxidation is the main effect studied, but the tests are based on colour reactions. Methanol extracts of *Phlomis fruticosa* and *P. lanata* have shown antioxidant activity. They prevent bleomycin-Fe (II)-catalysed superoxidation of arachidonic acid [3]. Forsythoside B and verbascoside (acteoside), the two major phenylethyl alcohol glycosides in the genus *Phlomis*, isolated from the methanol extract of the aerial parts of *P. caucasica*, were found to possess potent antioxidant properties against 2,2-diphenyl-1-picryl hydrazyl (DPPH) radical [4]. Using antioxidant activity evaluation models such as studies on butylated hydroxyanisole (BHA), 2,2′-azino-bis(3-ethylbenzothiazoline-6-sulfonic acid) (ABTS), DPPH, iron chelating properties, reducing ability, xanthine oxidase inhibition, superoxide radical scavenging, etc., antioxidant activity was determined for many extracts and compounds from *Phlomis* species, incl. *P. herba-venti*, *P. nissolii*, *P. tuberosa*, etc. [2]. There are no reports on isolated hepatocytes to test antioxidant activity and cell protection capabilities. A small number of in vivo studies have been performed to reveal the pharmacological properties of the plants. *P. aurea* and *P. ocymifolia* have been recognized as having antidiabetic properties. The activity may be mainly due to their ability to protect the liver and the pancreas by reducing oxidative stress in diabetes or by stimulating the production of enzymes involved in glucose metabolism. The antihyperglycemic activity of *P. anisodonta* methanol extract (PAME) in a streptozotocin (STZ)-induced rat model of diabetes was proved [5]. Streptozotocin causes irreversible damage to pancreatic cells, causing degranulation and reduced insulin secretion. STZ-induced diabetes is characterized by severe weight loss and the presence of diabetic complications such as myocardial, cardiovascular, gastrointestinal, neural, renal and bladder dysfunction due to oxidative stress. Administration of PAME (400 mg kg^−1^) for 10 days showed a significant decrease in blood glucose, an increase in plasma insulin levels and a decrease in body weight loss in STZ-treated rats. The observed antihyperglycemic effect resulted from the ability of PAME to improve plasma iron levels, reduce hepatic lipid peroxidation and block oxidative stress by activating hepatic antioxidant enzymes. A significant increase in hepatic superoxide dismutase, catalase and glutathione peroxidase activity was observed in PAME-treated rats [5]. In a similar manner, the polyorgan insufficiency, caused by ingestion of CCl_4_ is thought to be the most complete model of hepatic injury, resulting in encephalopathy, kidney failure and oxidative stress [6]. Thus, CCl_4_ is considered appropriate to investigate liver damage and the hepatoprotective effect of substances, incl. extracts and natural compounds [7].

*Phlomis tuberosa* L. is widely used in Bulgarian folk medicine for the treatment of gastrointestinal disorders such as gastritis, colitis, ulcer, hepatitis, cirrhosis, as well as kidney diseases. The aerial part of the species is used traditionally in the form of infusion. The species accumulates mainly flavone glycosides, iridoids, essential oil, phenylethanoid glycosides (mainly verbascoside), etc. [2,8]. The extract from the plant has been proved to possess anti-inflammatory activity in mice, compared to indomethacin [9]. There are no reports on the possible antioxidant and hepatoprotective activity of this plant. The aim of the study was to evaluate the defatted methanol extract of *P. tuberosa* (in vitro/in vivo) and the phenolic fraction, obtained from this extract (in vitro), in a carbon tetrachloride (CCl_4_)-induced hepatotoxicity model.

## 2. Results

### 2.1. Preparation of E and FF and Phytochemical Analysis

From the plant material, 40.68 g defatted extract from *P. tuberosa* (E) were obtained. After column chromatography purification, the resulting phenolic fraction (FF) was 2.08 g. Three HPLC methods with ultraviolet (UV) detection were used to determine their phytochemical composition and to perform quantitation by verbascoside. In addition, a spectrophotometric assay was performed to quantify the total polyphenols in the samples.

#### 2.1.1. Total Flavonoid Content

Using a modified pharmacopoeial method [10], the total flavonoid content of E was calculated as 3.50%, and FF, 19.22%, expressed as flavone glycosides. Representative chromatograms are presented in Figures S1 and S2.

#### 2.1.2. Identification of Compounds in E and FF

The qualitative method was validated according to [11]. Reagent specificity was tested by a blank solution. After analysis of the chromatogram, there were no peaks corresponding to the corresponding retention time of the standards. Each standard solution (see Section 4) was analysed by several sequential injections to examine sensitivity. In addition, model mixtures were prepared by mixing equal volumes of the standard solutions used. The RSD in the retention time of each analyte was less than 0.5 min, which is considered optimal [12]. Selectivity was examined as the SD of three subsequent injections of each standard solution, mixed with one of the sample solutions. The AUC of the corresponding compound rose, but the retention time remained with less than a 1% deviation from the mean value. The standard deviation (SD) in the absorbance units (AU) and the relative SD (%) were found to be ±1.0% when model samples were diluted 1:20 with MeOH and then injected three times. The limit of detection (LOD) was 0.001 mg/mL, calculated by the signal-to-noise ratio. The cut-off limit was less than 0.002 mg/mL. The recovery of twenty positive samples for each flavonoid was 98%. The rate of false negative and false positive responses was less than 2%. In addition, the UV spectra of each compound was in good correlation with the literature [13]. Ruggedness was examined by three days injection of the model solutions three times daily. Each response had an RSD less than 3%. The compounds identified in E were luteolin, quercetin, spiraeoside, apigenin-7-glucoside, luteolin-7-glucuronide and verbascoside (Figure S3, Table S1). The same compounds were proved in FF as well. As seen from the total flavonoids content, the quantity was different in E and FF, despite having the same composition.

#### 2.1.3. Validation of the Quantitative Analysis Method and Assay of Verbascoside

Verbascoside was quantified in both E and FF by a validated HPLC-UV method according to the approved International Conference of Harmonisation (ICH) Q2 (R1) guidelines [14]. Verbascoside had t_R_ = 13.17 min in the gradient program used (see Section 4). The method was validated according to the ICH. The system’s suitability, precision, linearity, accuracy, and selectivity were evaluated during method validation. Prior to performing the validation, the system was found to be suitable, as requested by examining the following criteria: the blank baseline had no interfering peaks above the 0.05% level at the retention time of verbascoside. A 0.15% standard solution of verbascoside (in MeOH) had a signal-to-noise ratio (S/N) ≥ 10. The standard solution agreement was≤ 1.9%. The resolution factor between neighbouring peaks was≥ 1.1 and the difference between two injections was ≤2.0%. 

##### Specificity

No significant interfering peaks (peak area > 0.1%) were observed at the retention time of verbascoside in a blank solution (MeOH). Using peak purity analysis, no evidence of co-elution was found. 

##### Limit of Quantification and Limit of Detection

The limit of quantification (LOQ) and limit of detection (LOD) were calculated from the standard deviation and slope of the response, using the signal-to-noise ratio. The LOQ for verbascoside was found to be 0.01 mg/mL, while the LOD was 0.001 mg/mL. 

##### Linearity/Accuracy

Linearity for verbascoside was evaluated from 25% to 150% of the nominal concentration. Linear regression was used to process the calibration data. The correlation coefficient of linearity was 0.9933 for verbascoside, which indicated a good correlation between the peak areas and the range of concentrations studied. The calibration curve is presented in Figure S4. The equation was y = 6942.2x + 1895.6. A linear least square analysis of the data gave a correlation coefficient (*r^2^* = 0.9933). The *y*-intercept obtained was≤ 0.5% of the 100% level, indicating that there is no significant bias for quantification. The method was considered accurate for verbascoside as per the ICH guidelines [14]. 

##### Precision

The method’s precision was evaluated by analysing six solutions of verbascoside at 100%. The average purity was 99.11% and RSD = 0.81% (*n* = 8), thus meeting the criteria of RSD [14]. 

##### Application of the Developed and Validated Method

The developed method was applied for the identification and quantitation of verbascoside. The result is presented in Figure S5. It was found that E had 5.9% (*w*/*w*) and FF, 32.4% (*w*/*w*) verbascoside.

#### 2.1.4. Total Polyphenols as Gallic Acid Equivalents (GAE)

Using a spectrophotometric assay with the Folin–Ciocalteu reagent, the quantity of total polyphenols was determined in both the E and the FF. It was found that E had 283.2 mg/g total polyphenols, and FF–812.7 mg/g total polyphenols, expressed as GAE [15]. The control solution of pyrogallol (0.1 mg/mL) had 332.9 mg/g GAE (Figure S6 and Table S2).

### 2.2. Pharmacological Evaluation

#### 2.2.1. Results from the In Vitro Experiments on Hepatocytes

**Figure 1:**
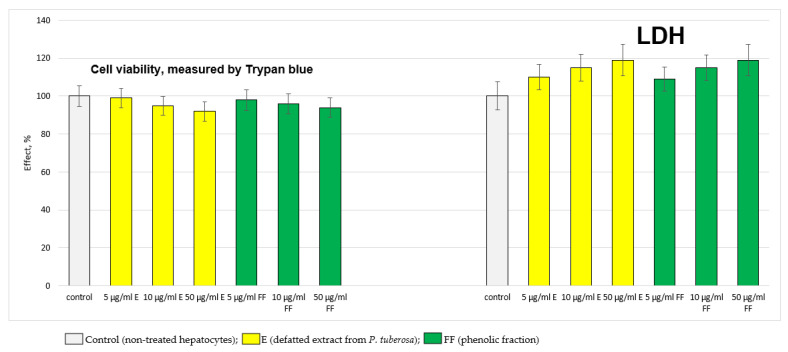
Effects of E and FF administered alone on hepatocyte viability (%) and LDH activity. LDH—lactate dehydrogenase activity.

**Figure 2:**
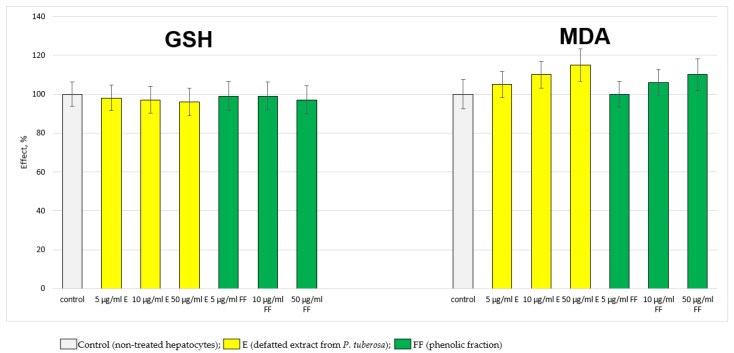
Effects of E and FF administered alone on GSH level and MDA production. GSH—level of reduced glutathione; MDA—malondialdehyde level.

The extract (E) and the phenolic fraction (FF), administered alone, did not exhibit a statistically significant hepatotoxic effect on the isolated hepatocytes. They did not change the hepatocyte viability, lactate dehydrogenase (LDH) enzyme activity, reduced glutathione (GSH) level and malondialdehyde (MDA) production (Figure 1 and Figure 2).

**Figure 3:**
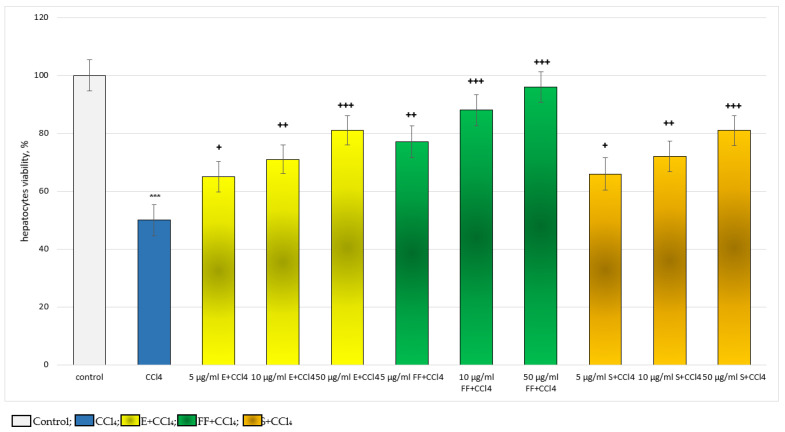
Effects of E, FF and S in a model of CCl_4_-induced hepatotoxicity, on hepatocyte viability. *** *p* < 0.001 vs. control (untreated hepatocytes); + *p* < 0.05; ++ *p* < 0.01; +++ *p* < 0.001 vs. CCl_4_. E—extract of *P. tuberosa*, FF—phenolic fraction, S—silymarin.

**Figure 4:**
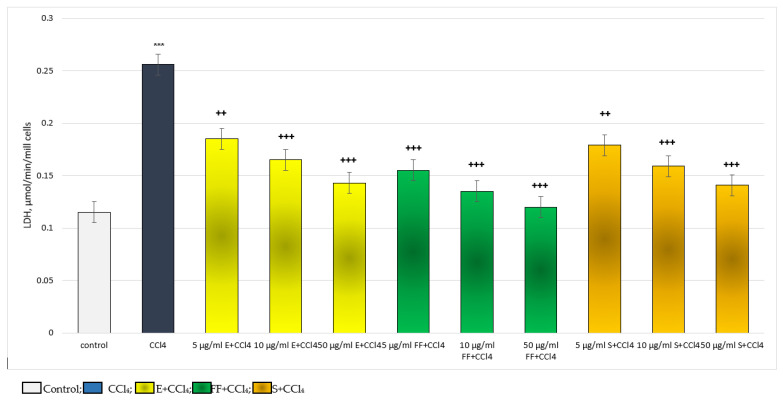
Effects of E, FF and S in CCl_4_-induced hepatotoxicity model on LDH activity. *** *p* < 0.001 vs. control (untreated hepatocytes); ++ *p* < 0.01; +++ *p* < 0.001 vs. CCl_4_. E—extract of *P. tuberosa*, FF—phenolic fraction, S—silymarin.

**Figure 5:**
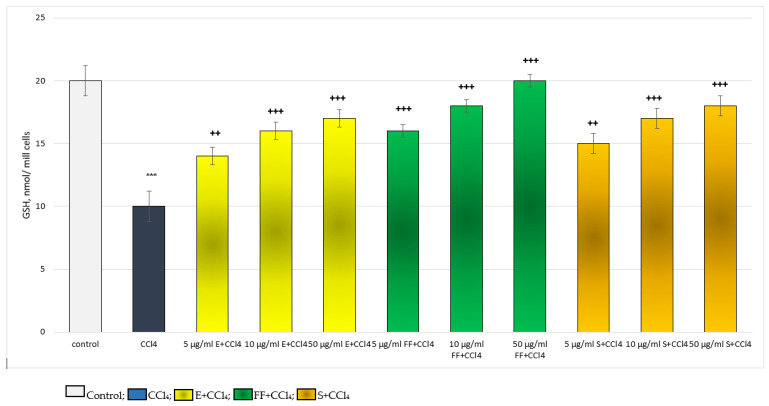
Effects of E, FF and S in CCl_4_-induced hepatotoxicity model, on GSH level. *** *p* < 0.001 vs. control (untreated hepatocytes); ++ *p* < 0.01; +++ *p* < 0.001 vs. CCl_4_. E—extract of *P. tuberosa*, FF—phenolic fraction, S—silymarin.

**Figure 6:**
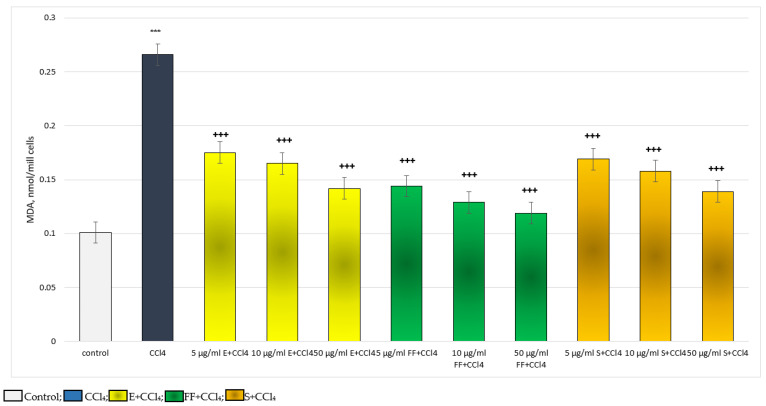
Effects of E, FF and S in CCl4-induced hepatotoxicity model on MDA production. *** *p* < 0.001 vs. control (untreated hepatocytes); +++ *p* < 0.001 vs. CCl_4_. E—extract of *P. tuberosa*, FF—phenolic fraction, S—silymarin.

In the carbon tetrachloride (CCl_4_)-induced metabolic bioactivation model, the effects of E and FF were compared with those of silymarin (S), a classic hepatoprotector and antioxidant. The substances were applied in concentrations of 5, 10 and 50 µg/mL. Administered alone, CCl_4_ exhibited remarkable, statistically significant, cytotoxic and pro-oxidant effects on isolated hepatocytes. It reduced hepatocyte viability and GSH level by 50%, increased LDH release by 123% and stimulated MDA production by 163%, relative to the control (untreated hepatocytes) (Figure 3, Figure 4, Figure 5 and Figure 6).

For hepatocyte viability, in a model of CCl_4_-induced hepatotoxicity, E, FF and S exhibited a concentration-dependent, statistically significant, hepatoprotective effect. The effect was most pronounced at the highest concentration of 50 µg/mL (Figure 3). Five µg/mL E preserved vitality by 30%; ten µg/mL by 42%; and 50 µg/mL by 62%, compared to the toxic agent. Five µg/mL FF preserved vitality by 54%; 10 µg/mL by 76%; and 50 µg/mL by 92%, compared to the toxic agent. Five µg/mL S preserved viability by 32%; 10 µg/mL by 44%; and 50 µg/mL by 62%, compared to the toxic agent. The effects of S and E on this indicator were comparable. The phenolic fraction exhibited a more pronounced effect than the extract and silymarin (Figure 3).

On the release of the LDH enzyme, in a model of CCl_4_-induced hepatotoxicity, E FF and S exhibited a concentration-dependent, statistically significant, protective effect. The effect was most pronounced at the highest concentration of 50 µg/mL (Figure 4). Five µg/mL extract reduced LDH release by 28%; 10 µg/mL by 36%; and 50 µg/mL by 44%, compared to the toxic agent. Five µg/mL FF reduced LDH release by 39%; 10 µg/mL by 47%; and 50 µg/mL by 53%, compared to the toxic agent. Five µg/mL S reduced LDH release by 30%; 10 µg/mL by 38%; and 50 µg/mL by 45%, compared to the toxic agent. The effects of S and E on this indicator were comparable. The phenolic fraction exhibited a more pronounced effect than the extract and silymarin (Figure 4).

For the level of GSH, in a model of CCl_4_-induced hepatotoxicity, E, FF and S exhibited a concentration-dependent, statistically significant, hepatoprotective effect. The effect was most pronounced at the highest concentration of 50 µg/mL (Figure 5). Five µg/mL E preserved GSH level by 40%; 10 µg/mL by 60%; and 50 µg/mL by 70%, compared to the toxic agent. Five µg/mL FF preserved GSH level by 60%; 10 µg/mL by 80%; and 50 µg/mL with 100%, relative to the toxic agent. Five µg/mL S preserved the GSH level by 50%; 10 µg/mL by 70%; and 50 µg/mL by 80%, compared to the toxic agent. The effects of the extract and silymarin on this indicator were comparable. The phenolic fraction exhibited a more pronounced effect than the extract and silymarin (Figure 5).

In terms of the production of MDA, a marker of lipid peroxidation in a model of CCl_4_-induced hepatotoxicity, E, FF and S exhibited a concentration-dependent, statistically significant, antioxidant effect. The effect was most pronounced at the highest concentration of 50 µg/mL (Figure 6). Five µg/mL E reduced MDA production by 34%; 10 µg/mL by 38%; and 50 µg/mL by 46%, compared to the toxic agent. Five µg/mL FF reduced MDA production by 46%; 10 µg/mL by 52%; and 50 µg/mL by 55%, compared to the toxic agent. Five µg/mL S reduced MDA production by 36%; 10 µg/mL by 41%; and 50 µg/mL by 48%, compared to the toxic agent. The effects of S and E on this indicator were comparable. The phenolic fraction exhibited a more pronounced effect than the extract and silymarin (Figure 6).

#### 2.2.2. Results from the In Vivo Experiment in Rats

**Figure 7:**
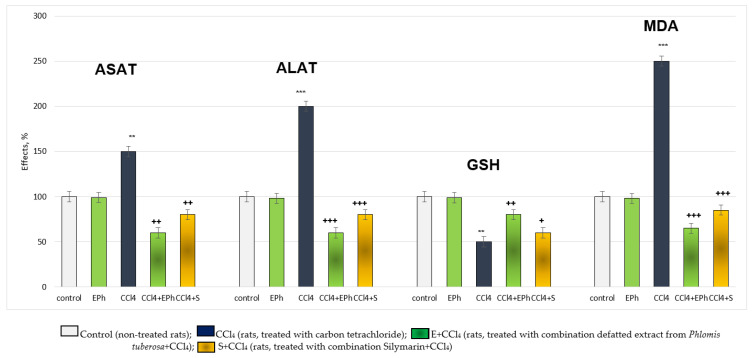
In vivo effects of E and S administered alone and in conditions of CCl_4_-induced hepatotoxicity. ^**^ *p* < 0.01; ^***^ *p* < 0.001 vs. control (non-treated rats); ^+^ *p* < 0.05; ^++^ *p* < 0.01; ^+++^ *p* < 0.001 vs. CCl_4_.

The approved experimental model led to no deaths during treatment. It was found that in vivo, administered alone, the extract did not reveal a statistically significant hepatotoxic effect. It did not change the serum levels of ALAT and ASAT, or the MDA production and GSH level. Administered alone, CCl_4_ revealed a statistically significant hepatotoxic effect by increasing the serum levels of ALAT and ASAT, as well as MDA production and decreasing the GSH level in liver homogenate. In combination with CCl_4_, the extract showed a statistically significant hepatoprotective effect on the examined biochemical parameters by decreasing the serum levels of ALAT and ASAT, preserving the GSH level and decreasing the MDA level. The effects of the extract on these biochemical parameters were stronger than the effects of silymarin (Figure 7).

**Figure 8:**
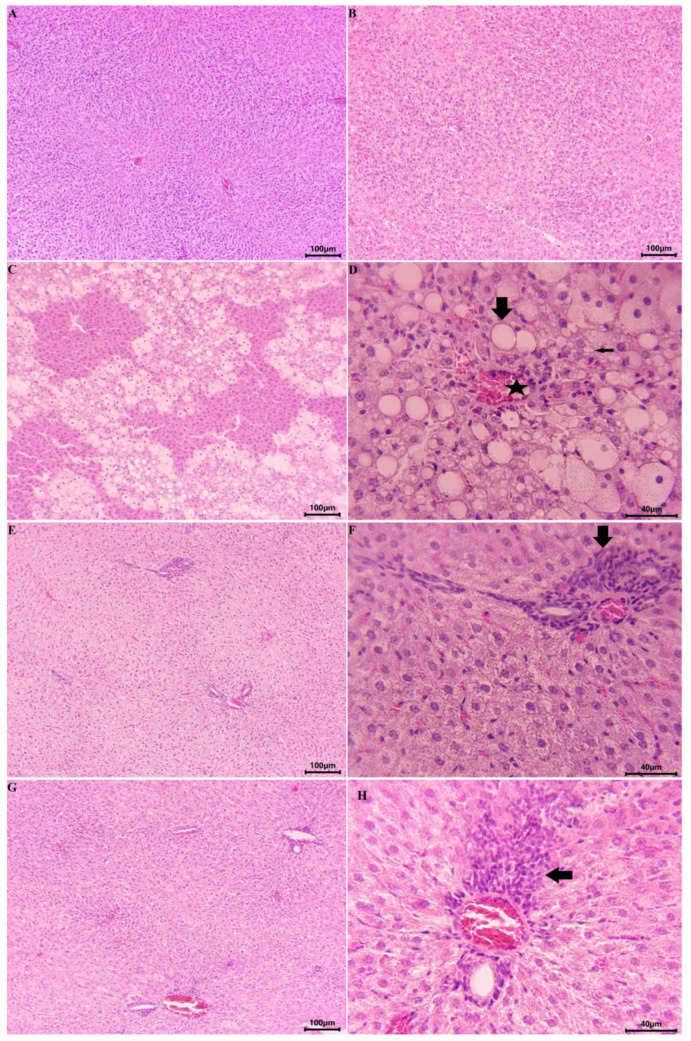
Histological examination of rat livers (H&E). (**A**) Control group of animals: normal histoarchitecture of liver parenchyma (100×); (**B**) Animals, treated with E: unaltered liver parenchyma, no microscopically visible alterations (100×); (**C**) CCl_4_-treated animal: diffuse areas of lipid degeneration (100×); (**D**) CCl_4_-treated animal: Presence of microvesicular (thin arrow) and macrovesicular (thick arrow) lipid degeneration and necrotic changes of hepatocytes around a terminal hepatic venule (asterisk) (400×); (**E**) Animals treated with CCl_4_ + E: unaltered microscopic structure of liver parenchyma (100×); (**F**) Animals treated with CCl_4_ + E: mononuclear accumulations (arrow) around portal tract (400×); (**G**) Animals treated with CCl_4_ + S: unaltered microscopic structure of liver parenchyma (100×); (**H**) Animals treated with CCl_4_ + S: mononuclear accumulations (arrow) around portal tract (400×). E—extract of *P. tuberosa*, S—silymarin.

A pathohistological examination of the livers was performed. In the control group, a normal histoarchitecture of the liver parenchyma detected lobules with centrally located terminal hepatic venules, surrounded with radially arranged hepatic lamellas and sinusoid capillaries (Figure 8A). In the animals of the extract-treated groups, the histological finding of the liver was the same as that of the control group, with no microscopically visible alterations (Figure 8B). In rats, treated with CCl_4_, diffuse lipid accumulations, accompanied by necrotic changes in liver structures, were microscopically observed (Figure 8C,D). A mixed type of lipid degeneration (micro- and macrovesicular) of the liver parenchyma was found. Pyknotic and lytic changes were observed in the nuclei and cytoplasm of some of the affected hepatocytes. The lesions were mainly of centrilobular localization, and bridging centro-central alterations were also observed. In some areas, some massive distributions of the lesions were detected. In the group of animals treated with CCl_4_ and E, an unaltered histoarchitecture of the liver lobules was detected (Figure 8E). A granular appearance of the cytoplasm and single vacuolar structures were found in some hepatocytes. Moderate accumulations of mononuclear cells were detected around some of the portal tracts (Figure 8F). A normal histological structure was observed in the liver tissue of animals treated with CCl_4_ and S (Figure 8G). In single areas, fine cytoplasmic granulation of the hepatocytes was observed. Limited mononuclear accumulations were observed around some portal tracts (Figure 8H).

## 3. Discussion

In experimental toxicology, in vitro systems have an important role in studying the metabolism of xenobiotics and establishing the possible mechanisms of toxic stress and the possibilities for its influence. Isolated hepatocytes are a suitable model for evaluating the cytotoxic and cytoprotective effects of some promising biologically active substances, newly synthesized or of biological origin.

One of the most important parameters to evaluate the metabolic capacity of a hepatocyte is the determination of vitality. This was performed with trypan blue dye. The chromophore in its molecule is negatively charged and does not react with cells whose membrane is intact but reacts with dead cells. Thus, cells that reject the dye are considered alive. A method for visual control of the permeability of the plasma cell membrane was proposed as early as the 1950s. The intact membrane is impermeable to trypan blue. The presence of a dye in the cytoplasm is an indicator of impaired integrity. This method is simple and allows, without special equipment, to obtain an answer about the functional state of the cell in a few minutes. The ability of nuclear proteins to adsorb the dye is also used for visual assessment of cell damage, and the faintest staining of the nucleus is indicative of a damaged cell membrane. Undamaged parenchymal cells have a yellow colour and a well-defined outline (due to membrane-retained trypan blue). They are clearly distinguishable from blue-stained dead cells [16].

When the plasma membrane is damaged, there is an outpouring of soluble enzymes from the cell. Lactate dehydrogenase, as well as liver transaminases, are the most characteristic representatives of the released enzymes in a membrane damaged due to cytotoxicity. Therefore, the ability of hepatocytes to release these enzymes is another important indicator for assessing the functional state of the cell membrane. The principle of determining LDH activity is based either on the oxidation of lactate to pyruvate or on the reduction of pyruvate to lactate. In the latter reaction, an equimolar amount of NADH is oxidized to NAD^+^. The oxidation of NADH is reflected in a decrease in the absorbance at 340 nm.

The most important protective mechanisms include the nucleophilic tripeptide glutathione, possessing a nucleophilic sulphur atom, which promotes the detoxification of reactive electrophiles in one of three ways: (1) through the formation of conjugates—a reaction catalysed by glutathione-S-transferase; (2) a chemical reaction with reactive metabolites until the formation of a conjugate without specific enzyme catalysis; (3) and as a proton and hydrogen atom donor for free radicals and reactive metabolites, respectively. It exists in two forms—reduced (GSH) and oxidized (GSSG). Glutathione is found everywhere in aerobic cells. The liver is among the organs with the highest glutathione content [16].

One of the main markers of lipid peroxidation is MDA. It is a product of lipid peroxidation, obtained from the breakdown of hydroperoxides formed during the oxidation of polyunsaturated fatty acids. The most studied aldehydes formed in the process of lipid peroxidation are: 4-hydroxynonenal, 4-hydroxyhexenal and MDA. MDA is a highly reactive metabolite and forms Schiff bases with the free amino groups of proteins and amino acids. It makes up about 2% of lipid peroxidation products formed, diffuses easily and has a longer life than free radicals. When it interacts with 2-thiobarbituric acid (TBA), a coloured complex consisting of one molecule of MDA and two molecules of TBA is formed. The intensity of the colour complex is proportional to the concentration of MDA [16].

There is a correlation between the amount of GSH in the cell and the production of MDA. In some cases, non-lipid oxidation causes a decrease in GSH, and conversely, because of GSH deficiency in the cell caused by the action of the reactive metabolite, a process of lipid peroxidation and an increased amount of MDA are observed [16].

Administered alone, the defatted extract and phenolic fraction obtained from *Phlomis tuberosa* did not exhibit hepatotoxic effects on all parameters, characteristic for the functional–metabolic status of isolated rat hepatocytes. Hepatotoxicity of CCl_4_ is perhaps the most widely studied compared to other toxic xenobiotics. This toxic agent causes centrilobular fatty degeneration and liver necrosis. As a fat-soluble compound, it is distributed throughout the body. Its chronic use causes liver cirrhosis and tumours, as well as kidney damage. Administration of low doses of CCl_4_ results only in fatty degeneration and the destruction of cytochrome P450 (CYP), which is mainly observed in the centrilobular zone of the liver [17]. CCl_4_ is bioactivated by CYP 1A2, CYP 2E1, CYP 2B1/B2 and CYP 3A. A trichloromethyl radical (CCl_3_•) is formed [18], which can participate in several reactions: (1) CCl_3_• can take a hydrogen atom from a suitable donor (for example, the methylene bridges in unsaturated fatty acids or from thiol groups) resulting in the formation of chloroform. Chloroform is a proven toxic metabolite of CCl_4_ under both in vitro and in vivo conditions. The other metabolic product is a lipid or thiol radical depending on the type of donor from which the hydrogen atom is taken. (2) The CCl_3_• can be oxidized to the trichloromethylperoxy (CCl_3_OO•) radical, with high reactivity. This radical subsequently produces phosgene. (3) The CCl_3_• free radical can bind covalently to microsomal proteins and lipids and thereby directly damage membrane phospholipids and cholesterol [19]. (4) The production of free radicals (CCl_3_•^−^ and CCl_3_OO•) unlock the process of lipid peroxidation. This mechanism destroys the cell membrane and the membranes of some vitally important organelles for the cell—the mitochondria and the endoplasmic reticulum. Some of the products of lipid peroxidation also damage the cell. Such products are 4-hydroxynonenal, MDA, conjugated dienes, and some other hydroxy alkenes. They inhibit protein synthesis and glucose-6-phosphatase activity. 4-Hydroxynonenal can react with the thiol groups of microsomal proteins, inhibit some enzymes or cause a decrease in GSH level [18]. An increase in membrane permeability was found in vitro. All the listed effects of the highly reactive aldehyde can also be caused by the parent compound. The most pronounced effect of 4-hydroxynonenal is that it blocks the storage of calcium in the endoplasmic reticulum and increases the level of free calcium in the cytosol.

The primary changes that occur after oral administration of a toxic dose of CCl_4_ are observed around the endoplasmic reticulum. One minute after its administration, it binds covalently to microsomal lipids and proteins in a ratio of 11:3. Conjugated dienes, indicators of lipid peroxidation, can be detected after 5 min. After 30 min, decreased protein synthesis, and changes in ribosomes and the endoplasmic reticulum are observed, which were demonstrated by electron microscopy. CYP content and activity are also reduced. Other indicators of damage to the endoplasmic reticulum are inhibition of the enzyme glucose-6-phosphatase and the disruption of calcium homeostasis. One to three hours after CCl_4_ administration, accumulation of triglycerides in hepatocytes in the form of fat droplets is observed. The loss of enzyme activity in the endoplasmic reticulum is enhanced. The level of cytosolic calcium increases, and vacuolization of the granular endoplasmic reticulum and ribosome impairment occurs. The plasma membrane deforms and ruptures, and lysosomes are released [17].

In vitro, in the CCl_4_-induced hepatotoxicity model, the phenolic fraction exhibited a more pronounced hepatoprotective effect compared to the extract and silymarin, a classic hepatoprotector and antioxidant.

Phenolic compounds, as well as silybin (a major constituent in the mixture, named silymarin), have been found to inhibit human CYP3A4 and CYP2C9, as well as the major hepatic glucuronosyltransferases [20]. On isolated hepatocytes, the better cytoprotective effect of the phenolic fraction in the CCl_4_ toxic model is most likely related to a synergistic effect leading to a change in the activity of some CYP isoforms involved in CCl_4_ bioactivation.

Silymarin has been found to protect the plasma membrane from CCl_4_-induced damage due to its antioxidant abilities and to modify the phospholipid content of the hepatocyte plasma membrane [21]. In 1994, the biochemical basis of the hepatoprotective action of silymarin was established. The authors proved that silymarin has three levels of action in experimental animals: (1) It acts as an antioxidant by trapping free radicals and increasing the intracellular concentration of the tripeptide glutathione; (2) it regulates the membrane permeability of the cell and increases its stability against damage by xenobiotics; and (3) at the nuclear level, silymarin increases ribosomal RNA synthesis by stimulating DNA polymerase I and affecting DNA transcription [22]. In 2003, silibinin (silybin A and B) was found to be metabolized by human microsomes to several metabolites. The main metabolite is demethylated silibinin and the other two metabolites are monohydroxy- and dihydroxy-silibinin. Silymarin has strong antioxidant, anti-inflammatory, cytoprotective and anticarcinogenic activity [23]. The hepatoprotective effect of *Phlomis tuberosa* extract and the phenolic fraction, as with the mechanism of silymarin, is most likely related to several possible mechanisms: cell membrane stabilization, preserving the level of GSH (the main scavenger of ROS) and the possible inhibition of some of the CYP isoforms involved in CCl_4_ metabolism, leading to the formation of reactive toxic metabolites.

In our study, the animal model is in very good correlation with other mammalian hepatic injuries, incl. human [6]. The results could easily serve as the basis for further evaluation on this extract as a powerful antioxidant and hepatoprotector. The quantitative methods applied are easily reproduced and suitable for routine quality control [12].

It is known that both verbascoside and flavonoids act in the same way a free radical scavengers [24]. The phenolic fraction was more active than the extract in the in vitro model because the flavonoid quantity and the amount of verbascoside were larger in the FF. In addition, the total antioxidant capacity of FF in vitro was in correlation with the total flavonoids and total polyphenols, expressed as GAE. The phenolic fraction exhibited a more pronounced effect than the extract and silymarin (see Section 2). Our results correlate with other data about the variety of protective effects of different species of *Phlomis*. In their experiments, Gu et al. [25] found good antioxidant activity, proved by ABTS, FRAP and DPPH in vitro, and a hepatoprotective effect of *P. maximowiczii* extracts on carbon tetrachloride-induced acute liver injury in mice. *P. stewartii* methanol extracts and *P. anisodonta* showed good antioxidant and anticancer activity and worked well to recover the tested clinical parameters in cigarette smoke/alloxan-induced diabetes animals, which indicated that the extracts also possess good antidiabetic, hepatoprotective and nephroprotective potential [26,27,28]. Antineurodegenerative, anti-inflammatory, antimicrobial and antioxidant activities were proved for extracts from *P. fruticosa* and *P. russeliana* [29,30,31,32]. Iridoid glucosides and triterpene acids from *P. linearifolia* revealed good hepatoprotective activity [33]. An antioxidant activity due to the phenolic content of *P. leucophracta* was proved [34]. For *P. Bovei De Noe* and *P. crinita*, antioxidant and antigenotoxic activities were found [35,36], and for *P. stewartii*, α-glucosidase inhibitory activity [37]. Phenylethanoid glycosides of *P. younghusbandii* ameliorate acute hypobaric hypoxia-induced brain impairment in rats [38]. *P. mauritanica* extracts reduce the xanthine oxidase activity, scavenge the superoxide anions and inhibit the aflatoxin B1-, sodium azide- and 4-nitrophenyldiamine-induced mutagenicity in bacteria [39]. Phenylpropanoids and flavonoids from *P. kurdica* were proved to be inhibitors of human lactate dehydrogenase [40]. Free-radical-scavenging activity for *P. caucasica* [4] and antioxidant effects for *P. lychnitis*, a traditional herbal tea [41,42], were proved.

This is in good correlation with our results on the phytochemical content of the extract of *P. tuberosa*; the high quantity of flavonoids and verbascoside is responsible, most likely, for the observed strong antioxidant effects.

## 4. Materials and Methods

### 4.1. Plant Material, Extraction, Preparation of the Phenolic Fraction and Phytochemical Analysis

The overground parts of *P. tuberosa* were collected from a cultivation field in Popica village, Bulgaria, in July 2022. The species was identified by one of us (I. K.) and a voucher specimen was deposited at the Herbarium of the Faculty of Pharmacy (FF-098). The air-dried plant material (400 g) was reduced to a coarse powder (3 mm), then percolated with dichloromethane (10 × 2 L) to remove the lipophilic constituents. The plant material was then aired and exhaustively extracted with 80% MeOH by percolation (24 × 2 L). The methanol extract was evaporated in vacuo and then lyophilized at −110 °C and 0.150 mbar for 96 h and named dried defatted extract (E). Nineteen grams of the resulting dried defatted extract were used to obtain the phenolic fraction. They were suspended in water, and separated on a Diaion HP-20 column, eluted with a gradient of H_2_O, 50% MeOH, 80% MeOH and 100% MeOH. The fractions obtained were lyophilized. The fraction eluted with 50% MeOH had the phenolic compounds (see Section 2). It was named phenolic fraction (FF) and was later used for the in vitro assay.

For the phytochemical analysis, commercially available flavonoids and verbascoside, as well as acetonitrile and methanol (HPLC gradient grade) were purchased from Sigma-Aldrich (St. Louis, MO, USA). *Ortho*-phosphoric acid, pyrogallol, sodium carbonate and Folin–Ciocalteu reagent were obtained from Merck (Darmstadt, Germany).

An HPLC system Young Lin 9100 (Hogye-dong, Anyang, Republic of Korea), equipped with YL 9101 vacuum degasser, YL 9110 quaternary pump, YL 9131 column thermostat, YL 9160 PDA detector, 7725i manual injector and Clarity software (v. 2) was used.

Total flavonoid content of E and FF was investigated by a method modified from the European Pharmacopoeia [10], using quercetin as the reference. Briefly, the samples were hydrolysed with HCl at 100 °C for 30 min. Then the hydrolysate was injected in the HPLC. The machine, the column, the solvents, as well as all other parameters were as reported [10]. Separations were monitored at 370 nm. Results were expressed as % flavonol glycosides.

For another two HPLC analyses, an aliquot (0.20 g) of each sample was dissolved in MeOH and diluted to 10.0 mL in a volumetric flask with the same solvent. Twenty µL were injected in the HPLC after membrane filtration (0.45 µm, PVDF).

For the qualitative analysis of flavonoids and verbascoside, the following gradient program was used: initial 5% B; from 5 to 13 min 5%→15% B (linear); from 13 to 45 min 15%→50% B (linear); from 45 to 50 min 50%→95% B (linear); from 50 to 55 min isocratic at 95% B; from 55 to 60 min back to 5% B (linear). Separations were performed on a pre-packed RP-C18 column Luna^®^ (100Å, 250 × 4.6 mm, 5 µm, Phenomenex, Torrance, CA, USA), coupled with Security Guard^®^ ODS cartridge. The column temperature was 35 °C. Solvents were H_2_O + 0.1% H_3_PO_4_ (A) and MeCN (B), filtered through membrane filter (0.45 µm, PVDF) prior to use. The flow rate was 1 mL/min. Chromatograms were recorded at 330 nm. Standard solutions of each compound were made in MeOH (0.25 mg/mL). The injection volume was 20 µL. The identification of flavonoids and verbascoside in the samples was performed based on their retention time coincidental to that of the authentic reference compounds. The method was validated by: sensitivity and specificity; selectivity (interferences), limit of detection, cut-off limit, unreliability region, rates of false positive and negative results; ruggedness [11]. The retention time was used as mean of three injections. To support identification, the PDA detector recorded the UV-vis spectra of the analytes, and it was compared to the reference UV-vis spectrum of each flavonoid [43].

For the quantitation of verbascoside, another gradient program was used: initial 10% B; from 5 to 25 min 10%→100% B (linear); from 25 to 28 min maintained at 100% B; from 28 to 30 min back to 10% B (linear). Separations were performed on Luna^®^ column (RP-C_18_ 100Å, 250 × 4.6 mm, 5 µm, Phenomenex, Torrance, CA, USA), coupled with Security Guard^®^ ODS cartridge. The column temperature was 35 °C and the solvents were H_2_O + 0.1% H_3_PO_4_ (A) and MeCN (B), filtered through membrane filter (0.45 µm, PVDF) before use. The flow rate was 1 mL/min and the analytical wavelength was 330 nm. Standard solutions of verbascoside were prepared in MeOH as follows 0.3, 0.6, 0.9, 1.2, 1.5 and 1.8 mg/mL. The injection volume was 20 µL. The method was validated as described by the International Conference of Harmonization (ICH 2005) [44] by specificity/selectivity; linear range (response and limits of detection and quantitation); accuracy and precision (sample spike and repeatability); and robustness.

The content of total polyphenols in E and in FF was analysed using a spectrophotometric method, using Folin–Ciocalteu reagent (FCR) [15]. Briefly, solutions of the E and E were prepared in H_2_O (1 mg/mL). Pyrogallol was used as the reference substance (0.1 mg/mL aqueous solution). Each sample (1 mL) was mixed with 5 mL ten-fold diluted FCR and 4 mL of 75 g/L aqueous solution of Na_2_CO_3_ were added. The mixture was left to stand in the dark for 30 min. After that, the absorbance was measured at 765 nm against H_2_O. Standard solutions of gallic acid were prepared as follows: 100, 150, 200, 250, 300, 350, 400, and 450 µg/mL in H_2_O. They were treated the same way as the samples and the calibration curve for gallic acid was constructed. The equation y = 0.0083 x + 0.0895 was obtained with a correlation coefficient *r^2^ =* 0.9931. The results for the total polyphenols in the samples were expressed as gallic acid equivalents (GAE, mg/g) [15].

Each experiment was conducted in triplicate. MedCalc 12.3 (MedCalc Software) was used. Results are presented as mean ± SD.

### 4.2. Animals

The research was conducted on a total of 30 male white Wistar rats (25 for the in vivo study and 5 for isolation of hepatocytes). The animals were obtained from the National Breeding Center of the Bulgarian Academy of Sciences, Slivnitsa, Bulgaria, and were reared under standard conditions in Plexiglas cages with free access to water and food and a 12 h/12 h light/dark regime at a temperature of 20–25 °C. Twelve hours before each study, the animals were deprived of their food. The experiments were conducted in accordance with Ordinance No. 15 on minimum requirements for the protection and humane treatment of experimental animals (No. 17, 2006) and the European Ordinance for working with experimental animals. The Institutional Ethics Committee at Medical University of Sofia (KENIMUS) gave clearance for the commitment of the experiment (No. 201, 2022). The animal experiments were approved with Permit No. 273, valid until 20 July 2025, issued by the Bulgarian Food Safety Agency.

### 4.3. Isolation and Incubation of Rat Hepatocytes

The primary hepatocyte suspension was obtained by in situ, two-step collagenase perfusion as described by Fau [16] with modifications made by us [45].

### 4.4. Incubation of Hepatocytes

Hepatocytes were pre-incubated for 30 min with concentrations of 5 µg/mL, 10 µg/mL, 50 µg/mL of E, and the FF, also with Silymarin, followed by a 1 h incubation in combination with CCl_4_. Silymarin, purity ≥ 97%, was obtained from Sigma-Aldrich (Germany). This is a mixture of antihepatotoxic flavonolignans from the fruit of *Silybum marianum*.

### 4.5. Determination of Hepatocyte Viability by Trypan Blue Test

A 0.05% trypan blue solution was prepared. Cells were counted under a microscope at 400× magnification [16].

### 4.6. Determination of the Activity of the Lactate Dehydrogenase Enzyme

After incubation with the substances (1.5 h), the hepatocytes were centrifuged at 400× *g* for 3 min. LDH was determined in the supernatant fluid. The sample was frozen at −20 °C. Immediately before the measurement, the sample was thawed and processed with the LDH kit (Werfen, Barcelona, Spain). The method is kinetic as the extinction was read spectrophotometrically at 340 nm at 30 s, 1, 2 and 3 min.

### 4.7. Determination of the Level of GSH

GSH was determined by measuring the non-protein -SH groups after precipitation of the proteins with trichloroacetic acid (TCA). Thiols present in the supernatant were determined using Ellman’s reagent (DTNB). A yellow colour formed at λ = 412 nm. After incubation, the cells were centrifuged at 400× *g* for 3 min. The supernatant was removed, and the precipitate was collected for GSH determination. It was treated with 5% TCA, then left for 10 min on ice, then centrifuged at 8000× *g* for 10 min (2 °C). The supernatant was collected for GSH determination and frozen at −20 °C. Immediately before measurement, samples were thawed and neutralized with 5N NaOH [16].

### 4.8. Determination of MDA Production

Twenty percent TCA was added to the hepatocyte suspension and the samples were homogenized. After homogenization, the samples were centrifuged for 5 min at 2000× *g*. After centrifugation, the supernatant was collected and 0.67% TBA was added. The samples were boiled at 100 °C for 30 min. After cooling, MDA was determined spectrophotometrically at λ = 532 nm [16].

### 4.9. Design of the In Vivo Experiments:

Each group consisted of five rats (*n* = 5). They were randomly allocated into five experimental groups:

Ist group: Control (NaCl 0.9% 14 days)
IInd group: *Phlomis tuberosa* extract (E) (100 mg/kg p.o./14 days) [46]
IIIrd group: CCl_4_ (10% solution, administered once on the 7th day; 1.25 mL/kg p.o.) [47]
IVth group: E (100 mg/kg p.o./14 days) in combination with CCl_4_ (10% solution, administered once on the 7th day; 1.25 mL/kg p.o.)
Vth group: Silymarin (S) (100 mg/kg p.o./14 days) in combination with CCl_4_ (10% solution, administered once on the 7th day; 1.25 mL/kg p.o.)

At the end of the experiment, blood was taken (see Section 4.11. Analysis of ASAT and ALAT in rat serum) and then the animals were sacrificed by decapitation.

### 4.10. MDA and GSH Measurement in Liver Homogenate

Livers were dissected and a small portion was taken for histopathology (see Section 4.12. Histopathological examination). The organs were stored oved ice before homogenization with a Teflon pestle using a tissue homogenizer Polytron (Kinematica AG, Switzerland) with the appropriate buffers. The MDA production and GSH level were measured spectrophotometrically at λ = 532 nm (for MDA) and λ = 412 nm (for GSH) according to [48].

### 4.11. Analysis of ASAT and ALAT

Before decapitation, blood was collected from the tail vein of each rat using a tube with EDTA. It was then centrifuged at 10,000× *g* for 10 min. The activities of aspartate aminotransferase (ASAT) and alanine aminotransferase (ALAT) were measured in the serum using an automated biochemical analyser BS-120 (Mindray, Shenzhen, China), with the appropriate kits, following the manufacturer’s instructions.

### 4.12. Histopathological Examination

Tissue was removed and fixed in 10% neutral buffered formalin. For the histopathological evaluation, slices with thicknesses of about 1 cm were washed under running tap water for 24 h, dehydrated continually with increasing concentrations of alcohol from 50% to 100%. The samples were then diaphonized in xylene and embedded in paraffin, melted in a thermostat at a temperature of 60 °C. Tissues were cut to 5 μm sections, deparaffinised and hydrated using xylene and graded alcohol concentration (to 50%), and stained with haematoxylin–eosin [49]. Microscopic examination and photography were performed with a Levenhuk D740T light microscope with an integrated camera.

### 4.13. Statistical Analysis

The results of the conducted experiments were processed through the statistical program ‘MEDCALC’, using the non-parametric Mann–Whitney method at significance levels of *p <* 0.05; *p* < 0.01; and *p* < 0.001.

## 5. Conclusions

An in vitro/in vivo hepatotoxicity and hepatoprotection evaluation of a defatted extract and a phenolic fraction, from *Phlomis tuberosa,* administered alone and in a carbon tetrachloride (CCl_4_)-induced metabolic bioactivation model, was performed. The extract and the phenolic fraction were analysed by three different HPLC methods. Several flavonoids as well as verbascoside were identified using reference substances. A quantitation using verbascoside was performed. In addition, total polyphenols were determined in the extract and in the phenolic fraction. Administered alone, the extract and the phenolic fraction had no concentration-dependent toxicity on hepatocytes. When CCl_4_ was added, both the extract and the phenolic fraction showed a concentration-dependent, statistically significant cytoprotective effect in vitro. The in vivo experiment was conducted in a CCl_4_-induced hepatotoxicity model in rats. Biochemical parameters, both in blood plasma and in liver homogenate (ASAT, ALAT, GSH and MDA) showed a statistically significant hepatoprotective effect of this extract. A histopathological study of the animals’ livers confirmed this effect. The findings were commensurable to that of silymarin.

## Data Availability

Data connected with this study are freely available from the corresponding author, upon reasonable written request.

## Supplementary Materials

The following supporting information can be downloaded at: https://www.mdpi.com/article/10.3390/ijms241310631/s1.
