# Supplementary material for: In Vitro/In Vivo Hepatoprotective and Antioxidant Effects of Defatted Extract and a Phenolic Fraction Obtained from Phlomis Tuberosa
Source: Int J Mol Sci. 2023 Jun 25;24(13):10631. doi: 10.3390/ijms241310631 (PMC10341447; doi:10.3390/ijms241310631)
Supplement: Supplementary file 1 [file ijms-24-10631-s001.zip › ijms-2452138-supplementary.pdf]

International Journal of
Molecular Sciences

MDPI

Article

In Vitro/In Vivo Hepatoprotective and Antioxidant Effects of Defatted Extract and a Phenolic Fraction Obtained from *Phlomis Tuberosa*

Magdalena Kondeva-Burdina [1],*, Aleksandar Shkondrov [2], Georgi Popov [3], Vasil Manov [3] and Ilina Krasteva [2]

[1] Laboratory of Drug Metabolism and Drug Toxicity, Department of Pharmacology, Pharmacotherapy and Toxicology, Faculty of Pharmacy, Medical University of Sofia, 2 Dunav st., 1000 Sofia, Bulgaria
[2] Department of Pharmacognosy, Faculty of Pharmacy, Medical University of Sofia, 2 Dunav st., 1000 Sofia, Bulgaria; shkondrov@pharmfac.mu-sofia.bg (A.S.); ikrasteva@pharmfac.mu-sofia.bg (I.K.)
[3] Department of Non-infectious Diseases, Pathology and Pharmacology, Faculty of Veterinary Medicine, University of Forestry, Sofia, Bulgaria; georgistpopov@yahoo.com (G.P.); manov_vet@abv.bg (V.M.)
* Correspondence: Correspondence: magdalenakondeva@gmail.com or mkondeva@pharmfac.mu-sofia.bg

Supplementary material

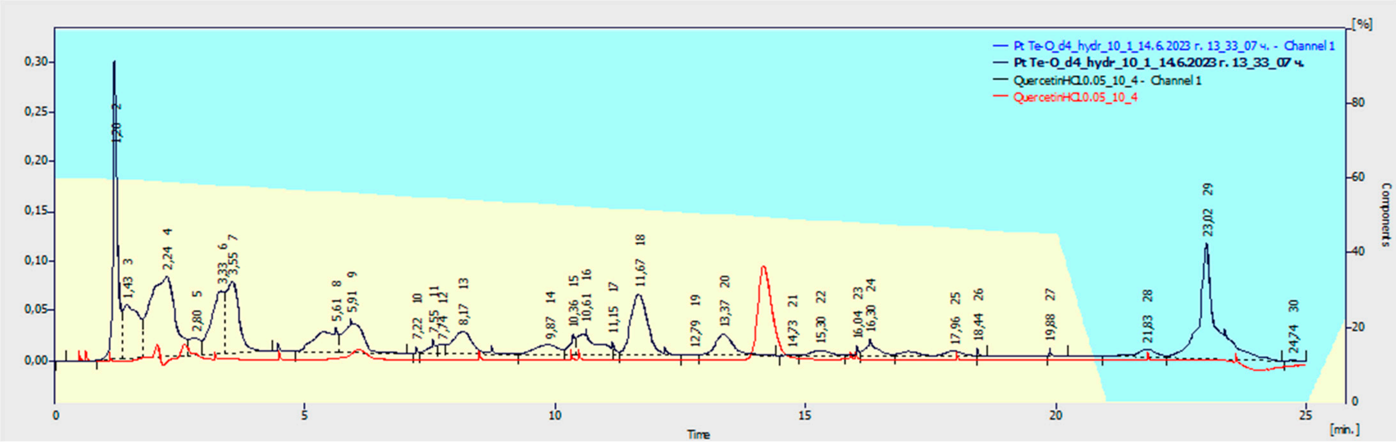

Figure S1. HPLC chromatogram of E used to perform quantitation of total flavonoids with quercetin reference

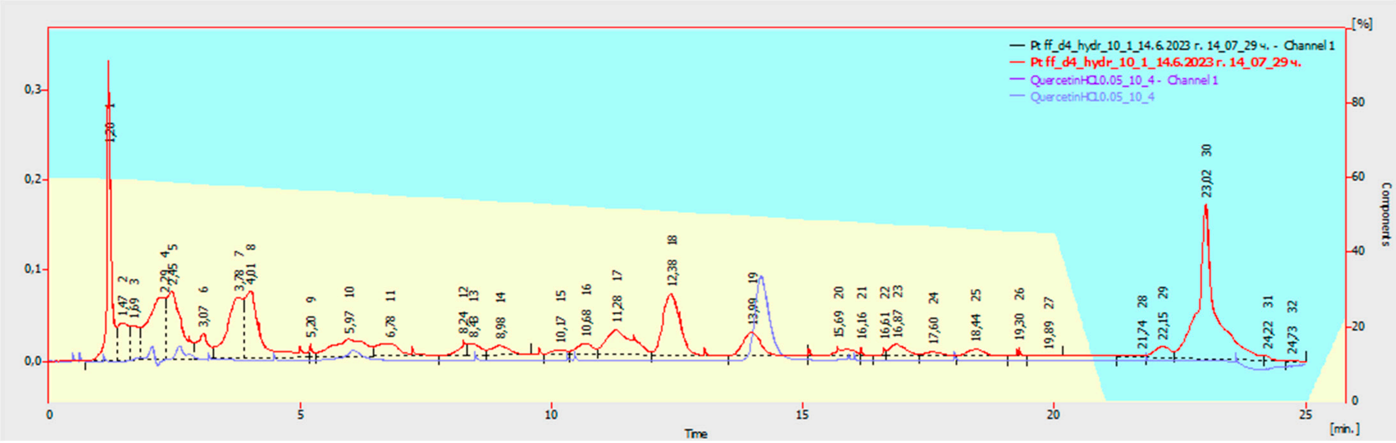

Figure S2. HPLC chromatogram of FF used to perform quantitation of total flavonoids with quercetin reference

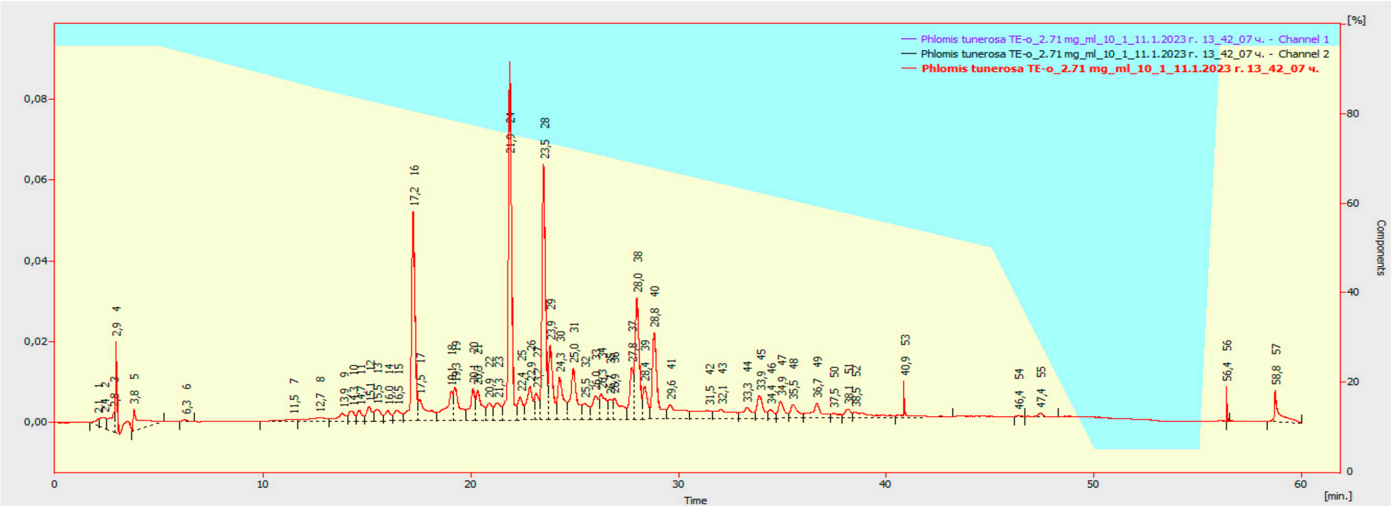

Figure S3. HPLC chromatogram of E used to identify the compounds

Table S1. Results from the quantitative analysis of E and FF

Copyright: © 2023 by the authors. Licensee MDPI, Basel, Switzerland. This article is an open access article distributed under the terms and conditions of the Creative Commons Attribution (CC BY) license (https://creativecommons.org/licenses/by/4.0/).

Compounds	t_R, min ± SD	E	FF
Rhamnetin	43.260 ± 0.18	-	-
Apigenin	37.830 ± 0.15	-	-
Luteolin	33.750 ± 0.12	+	+
Naringin	25.823 ± 0.10	-	-
Saponarin	21.227 ± 0.11	-	-
Verbascoside	24.470 ± 0.12	+	+
Apigenin-7-glucuronide	28.683 ± 0.13	-	-
Apigenin-7-glucoside	27.683 ± 0.12	+	+
Luteolin-7-glucuronide	25.740 ± 0.11	+	+
Luteolin-3’-glucuronide	26.810 ± 0.10	+	+
Spiraeoside	28.013 ± 0.13	+	+
Kaempferol-3-glucoside	27.210 ± 0.14	-	-
Quercetin-3-glucoside	25.157 ± 0.13	-	-

Vitexin	23.080 ± 0.12	-	-

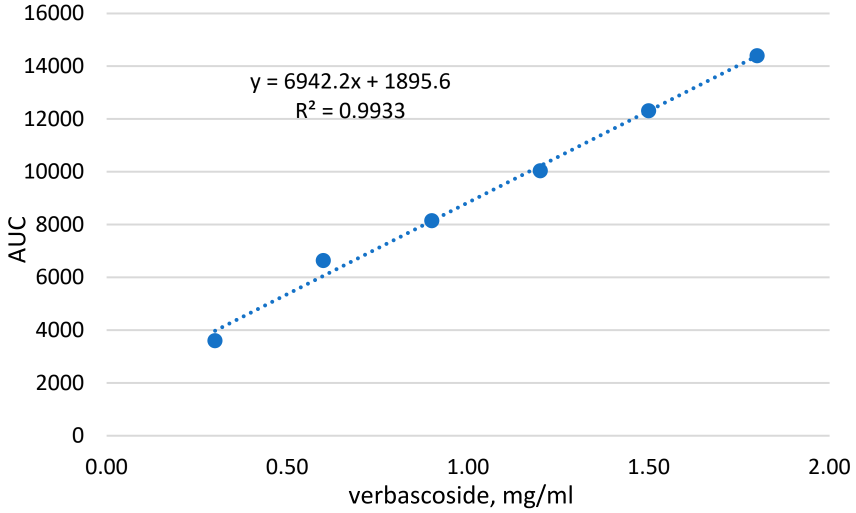

Figure S4. Calibration curve of verbascoside

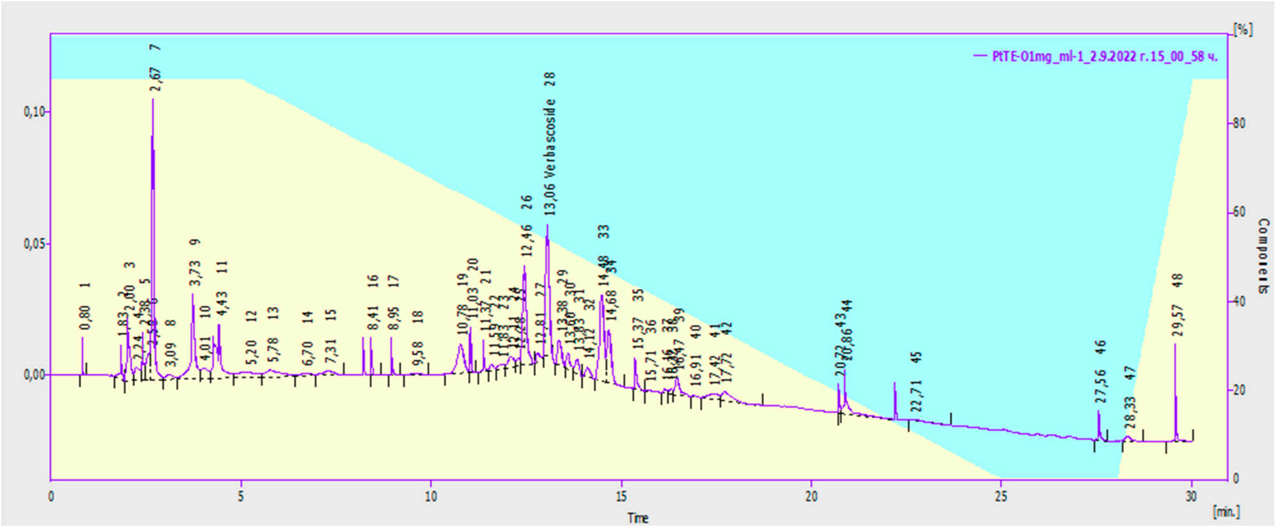

Figure S5. HPLC chromatogram of E used to preform quantitation of verbascoside

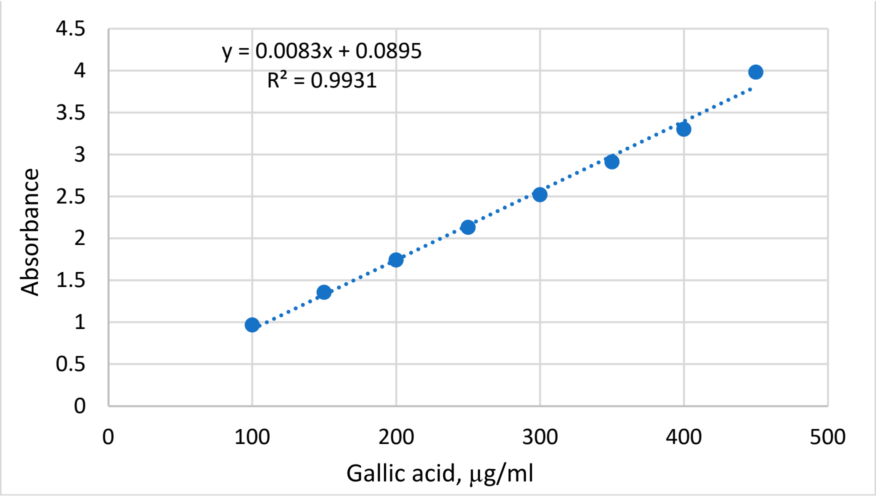

Figure S6. Calibration curve of gallic acid used to perform quantitation of total polyphenols in E and FF

Table S2. Total polyphenols in the samples

Sample	GAE ± SD, mg/g
E	283.2 ± 2.1
FF	812.7 ± 1.7
Control pyrogallol	332.9 ± 1.6

Disclaimer/Publisher's Note: The statements, opinions and data contained in all publications are solely those of the individual author(s) and contributor(s) and not of MDPI and/or the editor(s). MDPI and/or the editor(s) disclaim responsibility for any injury to people or property resulting from any ideas, methods, instructions or products referred to in the content.
